# Radiotherapy for the treatment of pulmonary hydatidosis in sheep

**DOI:** 10.1515/biol-2021-0139

**Published:** 2021-12-31

**Authors:** Yuefen Zhang, Pengfei Lu, Hongzhi Qi, Ge Wu, Rui Mao, Yongxing Bao

**Affiliations:** Department of Radiation Oncology, The First Affiliated Hospital of Xinjiang Medical University, No. 137, Liyushan South Road, Urumqi 830054, China

**Keywords:** radiotherapy, pulmonary hydatidosis, sheep, apoptosis, irradiation

## Abstract

Hydatidosis is an endemic disease causing a severe threat to public health. Drugs and surgery have been utilized for treatment, but their efficiency is not adequate. Therefore, new methods are required for treating such diseases. In this study, we attempt to evaluate the efficiency of radiotherapy for hydatidosis in sheep. The sheep naturally infected with pulmonary hydatid were randomly divided into four groups, including the control group subjected to no irradiation and the other three groups subjected to 30, 45, and 60 Gy irradiation, respectively. Gene expression of *caspase-3* and *gadd45a* and protein expression of BCL-2 and BAX in the lung tissues were evaluated after treatment. Our data showed that the irradiation with a dose of 30, 45, and 60 Gy significantly induced the expression of *caspase-3* and *gadd45a*. Immunohistochemical staining showed that the BCL-2 protein was downregulated after exposure to 45 Gy of irradiation, whereas the BAX expression was downregulated after irradiation at a dose of 45 and 60 Gy, respectively. On this basis, we speculated that 45 Gy might be a safe and effective dose for treating pulmonary hydatidosis in sheep, which induced lower expression of *caspase-3* and *gadd45a* in the cyst and a downregulation of BCL-2 and BAX in the adjacent lung tissues.

## 1 Introduction

Hydatidosis is an endemic disease in many countries, including South America, New Zealand, Canada, as well as Western China. At the adult stage, the *Echinococcus granulosus* lives in the intestine of carnivores, and the eggs are discarded in their feces. The eggs can survive for at least 1 year and could be infectious upon ingesting by an intermediate host such as sheep, goats, horses, or pigs, which finally grow into larvae within the bodies of these animals [1].

Lung is one of the most frequently involved organs by *E. granulosus* with an incidence of 10–40%, followed by the liver [2]. Patients newly infected by *E. granulosus* usually show small cysts and generally have no obvious symptoms. They are diagnosed occasionally during physical examination or chest X-ray scans. With the disease progression, some patients may present cough, sputum, chest pain, and hemoptysis combined with the enlargement of the cyst, causing compression, or inflammation [3].

The diagnosis of hydatidosis is mainly based on ultrasonography, X-rays, magnetic resonance imaging, and computed tomography (CT), as well as immunodiagnostic tests [4]. For the treatment of hydatidosis, patients are recommended to undergo surgery, chemotherapy, or observation. In a multicenter clinical study coordinated by the World Health Organization, benzimidazoles (BZD) such as albendazole and mebendazole are considered to be effective for treating hydatidosis [5]. To date, BZD and praziquantel are the major agents for antihydatid therapy. However, the response rates after administration of these agents are still not good due to a low blood drug concentration. In addition, there might be a high possibility of pulmonary hydatid rupture after chemotherapy [6]. It has been well acknowledged that surgery is still the main therapeutic approach for treating hydatidosis [7,8]. Nonetheless, many patients present recurrence after treatment.

Recently, our team has focused on the treatment of pulmonary hydatid infection using radiotherapy [9]. Our earlier studies indicated that radiotherapy contributed to the improvement of symptoms in those infected by pulmonary hydatid [10,11,12,13]. To date, there is still a lack of studies focusing on the molecular mechanisms of improvement of hydatidosis conditions after radiotherapy. In this study, we determined the gene expression of *caspase-3* and *gadd45a* that were tightly associated with apoptosis and cell death. Also, we determined the expression of BCL-2 and BAX serving as two important proteins involved in cell death and necrosis [14].

## 2 Materials and methods

### 2.1 Animals and study design

Twenty female sheep (3–5 years; body weight, 45 ± 10 kg) naturally infected with pulmonary hydatid confirmed by ultrasonography obtained from pastoral areas of Xinjiang autonomous region were reared in the experimental animal center of our hospital. The animals were fed in a temperature of 18–22°C and relative humidity of 40–60%. After CT confirmation, the animals were kept in the animal room for 1 week. All animals used in this study were euthanized using pentobarbital (85.8 mg/kg) and phenytoin (11 mg/kg) through intravenous injection. Death was declared with the presence of apnea and absence of audible heartbeat or corneal reflex as previously described [15].

Twenty sheep were randomly divided into the following groups: (i–iii) irradiation groups, subject to irradiation of 30 Gy (*n* = 5), 45 Gy (*n* = 5), and 60 Gy (*n* = 5), respectively, and (iv) control group received the same treatment except for irradiation.


**Ethical approval:** The research related to animal use has been complied with all the relevant national regulations and institutional policies for the care and use of animals. All study protocols were performed in line with the Ethics Committee of the First Affiliated Hospital of Xinjiang Medical University (approval No. IACUC-2014021002).

### 2.2 CT scan

After anesthesia, the sheep were fixed on an operating table using a thermoplastic membrane. Then CT scan was given using Big Bore Helical CT scanner (general electric). An experienced radiologist was responsible for depicting the irradiation area, and then the radiotherapy was performed by a professional radiotherapist. The radiotherapy was divided into three fractions with an interval of 2 days, and the predetermined dose was reached within 7 days. After radiotherapy, the cysts and the lung tissues adjacent to the endocyst were collected by biopsy for further detection.

### 2.3 Reverse-transcription quantitative PCR

Total RNA from endocyst was isolated using TRIzol (Takara Biotech, Dalian, China). The mRNA was reverse transcribed into cDNA by using a reverse transcriptase kit (Vazyme, China) according to the manufacturer’s protocols. Reverse transcript quantitative PCR (RT-qPCR) was performed on an Eppendorf MasterCycler platform (Wesseling-Berzdorf, Germany) using the SYBR Green system (Vazyme, China), and the gene transcription was normalized by β-actin. The relative expression level of each gene was calculated according to 2^−ΔΔCt^ method. The primers are listed in Table 1.

**Table 1:** Primers for RT-qPCR

Name	Sequence (5′–3′)
GADD45A F	TCGCTACATGGATCAGTGGG
GADD45A R	GTTGAACTCACTCAGCCCCT
caspase3 F	ATCCAGTCTTCCCTCCTT
caspase3 R	AGCACCGTTGTTTAGCAC
β-actin-F	CGCAAGTACTCCGTGTGGAT
β-actin-R	TAACGCAGCTAACAGTCCGC

### 2.4 Immunohistochemical analysis of lung tissue

The slices were deparaffinized with xylene and hydrated through a graded ethanol series (95, 90, 80, 70%, and pure water). After blocking with endogenous peroxidase, antigen retrieval was performed with sodium citrate. The sections were incubated with primary anti-Bax (1:50, Category No.: A7626, Bioss, China) and anti-BCL-2 antibody (1:400, Category No.: MG53719-CH, Bioss, China) at 4°C for overnight. Then the sections were incubated with an horseradish peroxidases-conjugated secondary antibody for 20 min at room temperature. After washing with phosphate buffer saline, the sections were incubated with 3,3′-diaminobenzidine, counterstained with hematoxylin, and promoted blue with blue liquid, followed by dehydration. Finally, the sections were cover-slipped with neutral plastic and observed under a microscope.

### 2.5 Statistical analysis

Data analysis was performed by one-way analysis of variance followed by Tukey’s multiple comparison tests using a GraphPad Prism 7 software (GraphPad Corporation). A *P* value of <0.05 was considered to be statistically significant.

## 3 Result

### 3.1 Conditions of hydatid cyst

**Figure 1:**
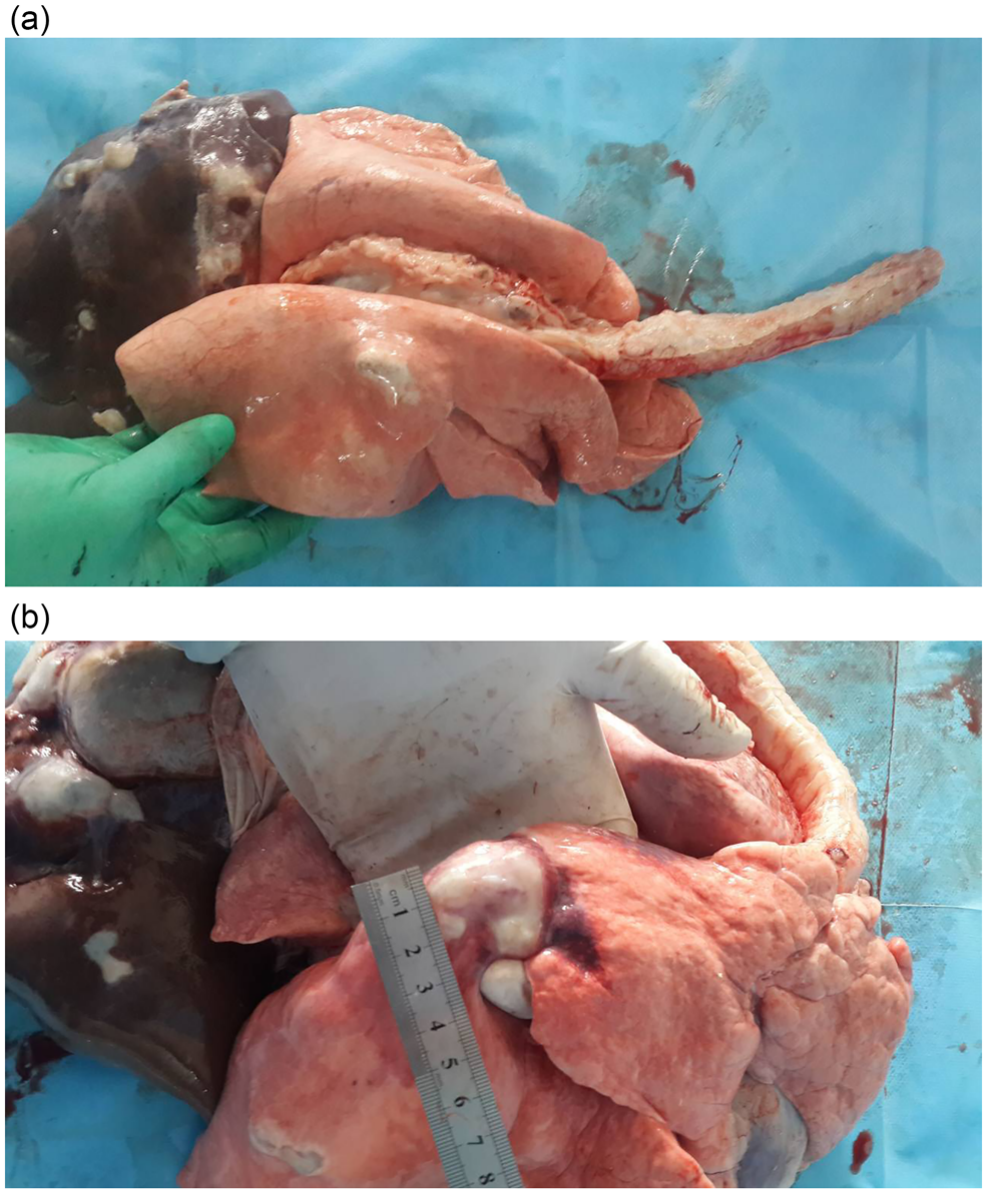
Morphology of cyst in the lung tissues in (a) control group and (b) radiation group.

The hydatid cysts in the lung tissues of sheep were multiple cysts of various sizes. The majority of lung tissues showed the presence of protoscoleces in the cysts (Figure 1). After radiation, the size showed a decline to some extent.

### 3.2 Irradiation induced the expression of *caspase-3* and *gadd45a* in the hydatid cyst

**Figure 2:**
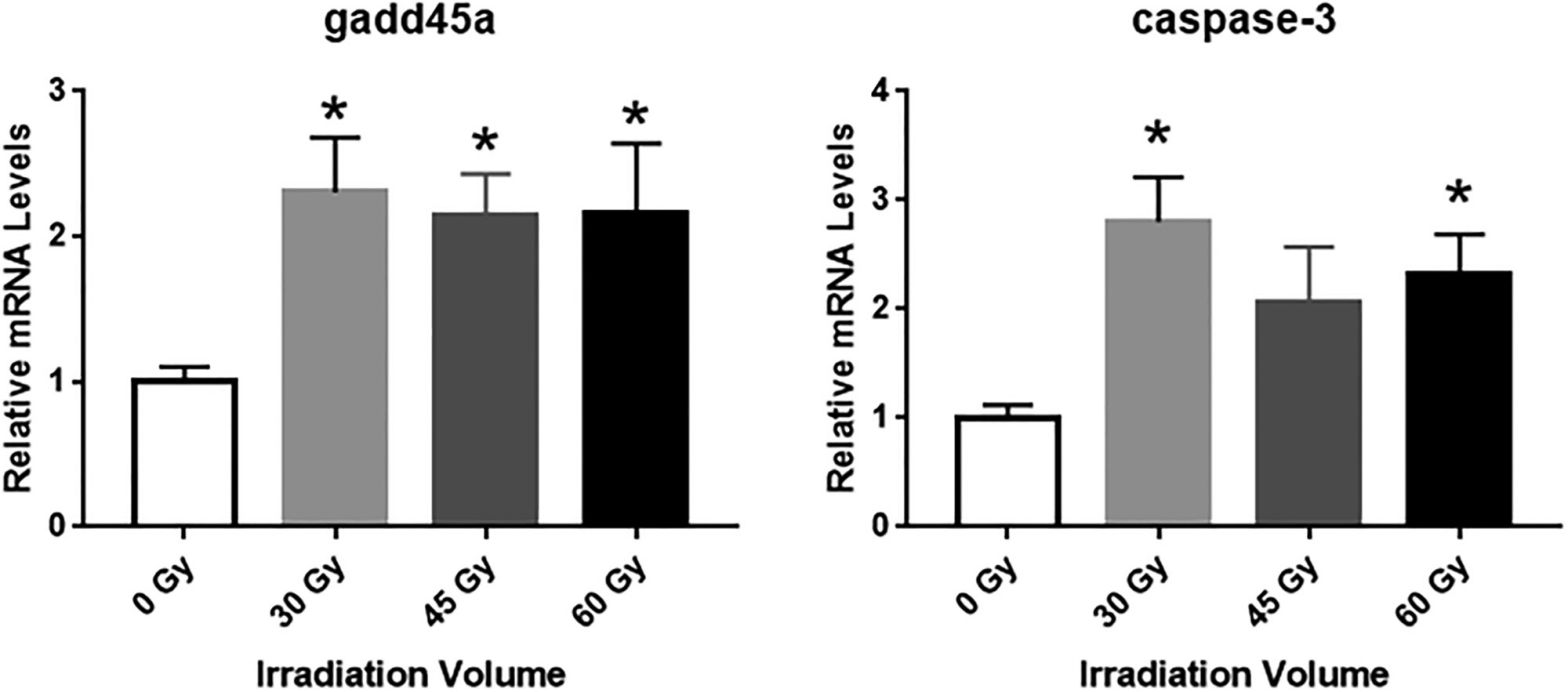
Effect of irradiation on the expression of *caspase-3* and *gadd45a*. The mRNA level of *gadd45a* and *caspase-3* was evaluated by using RT-qPCR (*n* = 5 in each group). The presented values are the means ± standard error mean. *P < 0.05, compared with control group.

To investigate the effects of irradiation on hydatid cysts, the expression levels of two key genes (*caspase-3* and *gadd45a*) involved in apoptosis and cell death were evaluated using RT-qPCR. The relative expression of *gadd45a* mRNA in the 30 Gy group was about 2.4-fold higher than in the control group. In addition, the expression of *gadd45a* mRNA in the 45 and 60 Gy groups was about 2.1–2.2 fold higher than that of the control group (Figure 2). For the expression of *Caspase-3* mRNA, its expression in the 30 Gy group was nearly 3.0-fold compared with that of the control, whereas the expression of *Caspase-3* mRNA in 45 and 60 Gy groups was 2.0-fold and 2.2-fold compared with that of the control group (Figure 2). All these indicated that irradiation caused apoptosis and necrosis in cystic cells.

### 3.3 Irradiation reduced the expression of BCL-2 and BAX in lung tissue involved by the cyst

**Figure 3:**
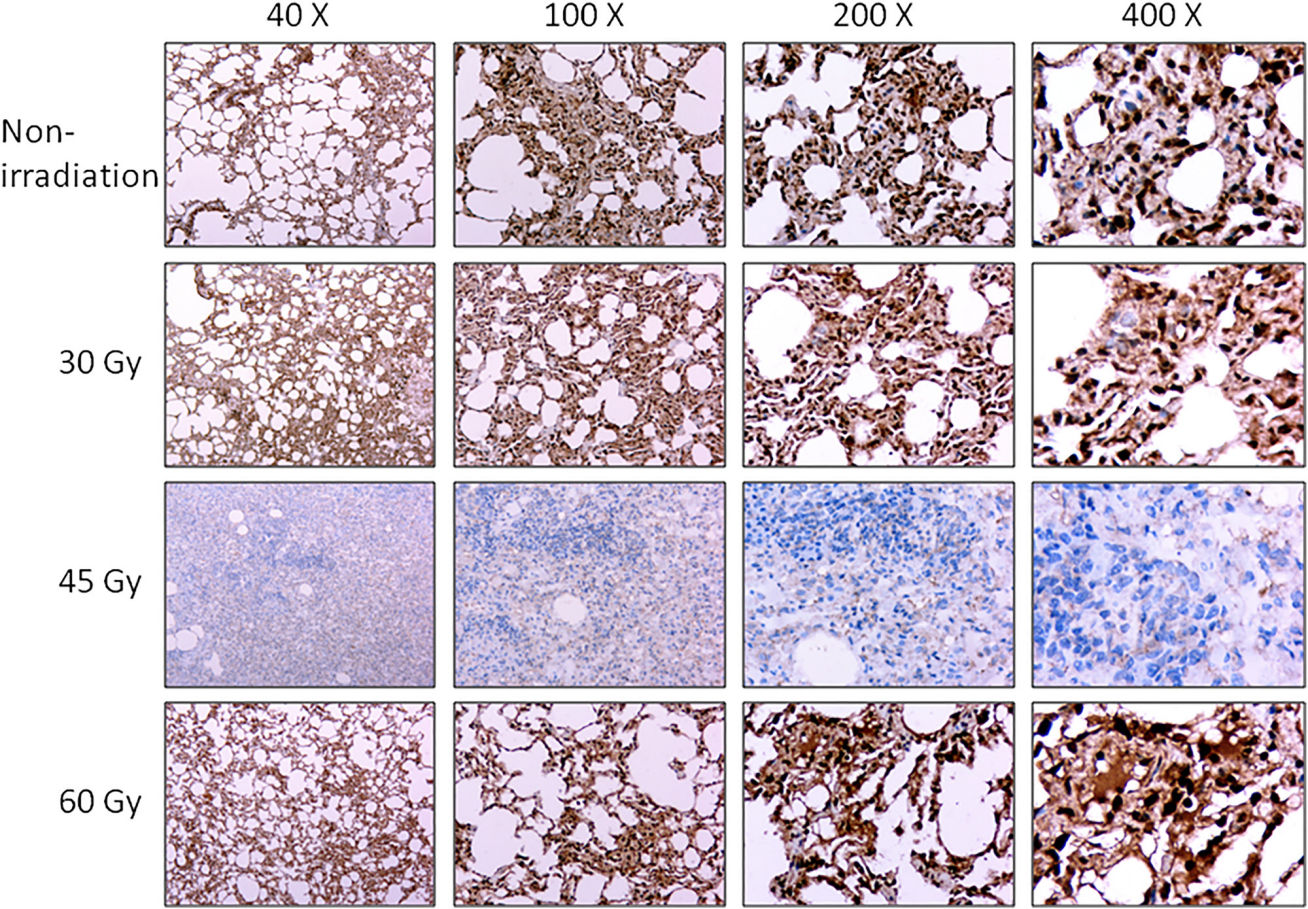
Effects of irradiation on the expression of BCL-2. The protein level of BCL-2 was detected by using immunohistochemistry under a magnification of 40×, 100×, 200×, and 400×, respectively.

**Figure 4:**
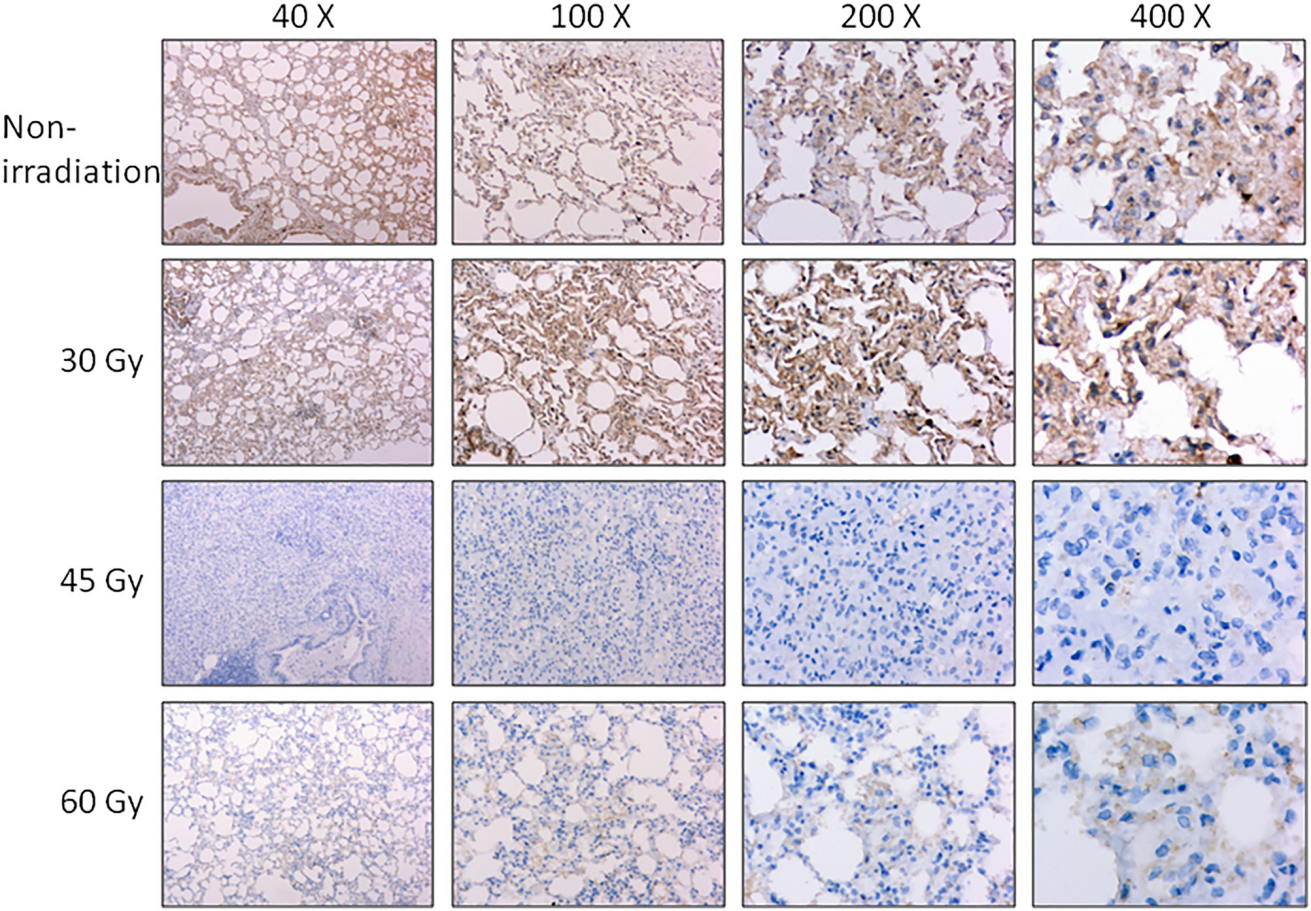
Effects of irradiation on the expression of BAX. The protein level of BAX was detected by using immunohistochemistry under a magnification of 40×, 100×, 200×, and 400×, respectively.

With the continuous growth of *E. granulosus*, the surrounding organs and tissues were compressed, resulting in cell death and tissue atrophy or necrosis, as well as final dysfunction [16]. In the present study, we detected the expression levels of BCL-2 and BAX in the lung tissue using immunohistochemistry, which showed no significant difference in the expression of BCL-2 after exposure to irradiation with a dose of 30 and 60 Gy, respectively. In contrast, the expression level of BCL-2 was significantly downregulated after exposure to a dose of 45 Gy (Figure 3). This indicated recovery from cell death induced by the cyst. BAX expression was significantly downregulated in the cyst after exposure to a dose of 45 and 60 Gy, rather than that of 30 Gy (Figure 4), indicating 45 and 60 Gy of irradiation reversed the cell death induced by the cyst.

## 4 Discussion

Hydatid disease is a serious zoonosis caused by *Echinococcus* hydatid parasitism in animal hosts, with a strong infectious potency to human individuals [17]. Hydatid disease has been considered to be an epidemic in the world. In recent years, extensive studies have been conducted to investigate the ecology and biological features of hydatid disease, and a comprehensive prevention system has been established [18,19,20]. This, to some extent, greatly promotes clinical diagnosis and treatment [21].

The progression of hydatid disease is prolonged, and some patients are asymptomatic for several years. The larva of hydatid acts as tumors that are slowly growing and gradually invading the organ. The severity of the disease may be closely related to the marked fibrosis of the tissue around the cysts. Due to hematogenous spread, it will gradually extend to the adjacent tissues and distant organs [22]. To date, the treatment of hydatid disease is still mainly reliant on the surgery and administration of agents (e.g., BZD). Recently, few studies have been available to focus on utilizing irradiation for treating such disease. In an earlier study, Zhao et al. indicated that heavy-ion radiation could induce the extinction of hydatid cysts *in vitro* [23]. However, rarely studies have been focused on the roles of radiation in hydatid disease under *in vivo* conditions [24].

The caspase family is a key element in the process of cell apoptosis. Its activation and overexpression could regulate cell apoptosis through interacting with multiple protein factors [25]. Caspase-3 is the main effector in the process of apoptosis [26]. At present, a large number of patients may present recurrence after surgery, administration of drugs, and other methods. To our best knowledge, irradiation can effectively kill the hydatid, causing relatively low injuries to normal tissues, which may serve as a safe and effective option for treating hydatidosis. In this study, we investigated the efficacy of radiation therapy for hydatidosis through detecting the apoptosis and cell death-related genes and proteins in the cyst and the adjacent lung tissue, respectively. Our data showed that irradiation could significantly induce the expression of *caspase-3* and *gadd45a*, serving as two important genes involved in apoptosis and cell death. This indicated that the irradiation could effectively kill the hydatid in the lung tissues of sheep.

The immunohistochemical staining showed that 30 Gy irradiation triggered no reduction in the expression of BCL-2 and BAX. However, a dose of 45 Gy significantly suppressed the expression of BCL-2 and BAX in the lung tissues involved by the cyst, suggesting beneficial effects on the host. Interestingly, 60 Gy irradiation did not reduce the protein level of BCL-2. This may be related to the fact that excessive irradiation causes injuries to the lung tissue, which indicates that the irradiation dose should be within the tolerance of normal tissues. Irradiation significantly enhanced the protein expression of BAX and BCL-2. This indicated that the killing effects of radiation on the hydatid disease were associated with the activation of the apoptosis pathway. Similarly, in an earlier study, irradiation could induce cell death by facilitating apoptosis [27].

There are limited reports about the treatment of hydatidosis based on irradiation, as most of the studies were carried out under *in vitro* conditions [28]. This is the first study to report the treatment of pulmonary hydatidosis in sheep by using irradiation, and we hope this may provide some insight for the clinical treatment. However, there are some limitations to this study. We only investigated the efficiency of radiation on treating hydatidosis through determining the expression of apoptosis-related markers. Little is known about the potential mechanism in it. Besides, the sample size is not large due to difficulty in sampling.

Three irradiation doses were used in this experiment, and the results showed that 45 Gy might be safe and effective for treating pulmonary hydatidosis in sheep. This was supported by an induction of *caspase-3* and *gadd45a* expression in the cyst and a downregulation of BCL-2 and BAX in the adjacent lung tissues.
